# CUMULATIVE (DIS)ADVANTAGE IN COGNITIVE HEALTH: RURAL/URBAN RESIDENCY, HUKOU, AND COGNITIVE AGING IN OLDER CHINESE

**DOI:** 10.1093/geroni/igac059.678

**Published:** 2022-12-20

**Authors:** Yuan Zhang, Qian Song, Jen-Hao Chen

**Affiliations:** University of North Carolina at Chapel Hill, Chapel Hill, North Carolina, United States; Universiy of Massachusetts Boston, Boston, Massachusetts, United States; National Chengchi University, Taipei City, Taipei, Taiwan (Republic of China)

## Abstract

Urban residency benefits cognition in later life. In China, the household registration system (hukou) creates another place-based advantage/disadvantage for cognitive aging. We exam the interplay of rural/urban residence and hukou across the life-course in creating cumulative advantage/disadvantage in cognitive aging trajectories using longitudinal data from the 2011-2018 China Health and Retirement Longitudinal Study and its Life History Survey. Results from linear mixed effect models show that always urban dwellers with urban hukou exhibited the highest level of cognitive function and the slowest decline, and the always rural dwellers with rural hukou had the worst cognitive function and experienced a faster decline. Rural-to-urban shifts had better cognitive health than always rural dwellers, but the advantages depend on urban hukou attainment and specific mechanisms. This research suggests cumulative advantage/disadvantage in cognitive aging by residence and hukou throughout life and demonstrates that institutional discrimination and exclusionary policy may create inequality in aging processes.

